# Supra- and sub-threshold intracellular-like recording of 2D and 3D neuronal networks using nanopillar electrode arrays

**DOI:** 10.1038/s41378-024-00817-y

**Published:** 2024-12-05

**Authors:** Shivani Shukla, Joshua L. Schwartz, Callum Walsh, Wen Mai Wong, Vrund Patel, Yu-Peng Hsieh, Chichi Onwuasoanya, Shaoming Chen, Andreas Offenhäusser, Gert Cauwenberghs, Francesca Santoro, Alysson R. Muotri, Gene W. Yeo, Sreekanth H. Chalasani, Zeinab Jahed

**Affiliations:** 1 Shu Chien-Gene Lay Department of Bioengineering, University of California San Diego, La Jolla, CA 92093 USA; 2 Aiiso Yufeng Li Family Department of Chemical and Nano Engineering, University of California San Diego, La Jolla, CA 92093 USA; 3 Department of Cellular and Molecular Medicine, University of California San Diego, La Jolla, CA 92093 USA; 4 Institute for Genomic Medicine, University of California San Diego, La Jolla, CA 92093 USA; 5 Molecular Neurobiology Laboratory, The Salk Institute for Biological Studies, La Jolla, CA 92037 USA; 6 Institute of Biological Information Processing—Bioelectronics, IBI-3, Forschungszentrum Jülich, Jülich, 52428 Germany; 7 Neuroelectronic Interfaces, Faculty of Electrical Engineering and IT, RWTH Aachen, Aachen, 52074 Germany; 8 Department of Pediatrics, University of California San Diego, San Diego, CA USA; 9 Center for Academic Research and Training in Anthropogeny (CARTA) and Archealization (ArchC), University of California San Diego, La Jolla, CA 92093 USA; 10 Sanford Stem Cell Education and Integrated Space Stem Cell Orbital Research (ISSCOR) Center University of California San Diego, La Jolla, CA 92093 USA; 11 Sanford Stem Cell Institute Innovation Center, University of California San Diego, La Jolla, CA 92093 USA; 12 Center for RNA Technologies and Therapeutics, University of California San Diego, La Jolla, CA 92093 USA

**Keywords:** Nanobiotechnology, Bionanoelectronics

## Abstract

The brain integrates activity across networks of interconnected neurons to generate behavioral outputs. Several physiological and imaging-based approaches have been previously used to monitor responses of individual neurons. While these techniques can identify cellular responses greater than the neuron’s action potential threshold, less is known about the events that are smaller than this threshold or are localized to subcellular compartments. Here we use NEAs to obtain temporary intracellular access to neurons allowing us to record information-rich data that indicates action potentials, and sub-threshold electrical activity. We demonstrate these recordings from primary hippocampal neurons, induced pluripotent stem cell-derived (iPSC) neurons, and iPSC-derived brain organoids. Moreover, our results show that our arrays can record activity from subcellular compartments of the neuron. We suggest that these data might enable us to correlate activity changes in individual neurons with network behavior, a key goal of systems neuroscience.

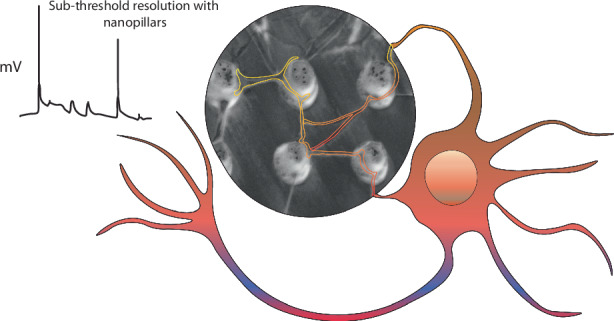

## Introduction

Nervous systems include both neuronal and non-neuronal cells, which are organized into anatomical and functional networks^1^. Many techniques have been developed to record activity from neurons, which are excitable cells. Specifically, a whole cell patch clamp preparation allows for changes in electrical or voltage to be monitored in individual neurons. This method led to the first quantitative recordings of action potentials^2,3^. A rapid initial movement of sodium followed by potassium ions across the membrane leading to change in voltage above a threshold has been defined as an action potential. To increase the throughput of this approach, arrays of electrodes have been used both in vitro and in vivo^4–7^. These arrays can record action potential events across hundreds of neurons and allow neuroscientists to link activity in individual neurons with entire networks. However, there have only been a few key examples within the past decade where electrode arrays have been able to record not only supra-threshold action potential signals, but also sub-threshold signals originating from subcellular events^8–10^. These previous studies offered a glimpse into methods to obtain “multiscale” electrophysiological recordings which tie subthreshold synaptic processing to network-level activity^11–13^. Moreover, this sub-threshold activity is closely linked to the metabolism and functional morphology of the neuron as sodium^14,15^, potassium^16^, calcium^17,18^, and other ion channels are differentially regulated in various subcellular compartments^19,20^. Recent advances in automated patch clamp methods have achieved subcellular and sub-threshold measurements, which do not always mimic neuronal networks found in vivo and are limited by throughput^21^. We have developed a platform to enable sub-threshold, subcellular, and intracellular-like recordings within intact neuronal networks.

Nanoelectrode arrays (NEAs) are arrays of nano-scale vertical conductive structures that have become promising tools for recording intracellular membrane potentials^8–10,22–24^. NEAs leverage the malleable nature of a cell’s plasma membrane to create a region of high-seal resistance, allowing for tight electrical coupling between the electrode and cell. Oftentimes spontaneously, but sometimes following a brief electrical pulse, the membrane is temporarily permeabilized, allowing the electrode to gain intracellular access. While the nature of these intracellular signals is poorly understood, an ensemble of proteins including integrins and curvature sensitive proteins allow the plasma membrane to adhere to the underlying nanostructures through curved adhesions^25^. A critical gap in NEA technology is the optimization of various nanostructures to target different kinds of cell sizes, morphologies, and structures. We optimize the electrode size, chemical functionalization and device packaging to achieve intimate interfaces with neurites in diverse neuronal cultures. Specifically, we designed nanopillar electrodes to possess the spacing and dimensions needed to physically interact with both neuronal somas and processes. Using two-step wet and dry etching processes on fused silica wafers, we constructed nanopillars with dimensions (pitch: 5 μm, diameter: 600 nm, height: 3 μm) previously shown to enhance cell engulfment^22,26^, as well as radii of curvature (1–3 μm^-1^) matching previously observed values which promote neurite outgrowth and guidance^27,28^. Secondly, we optimized the surface functionalization protocol needed to achieve adhesion of both primary and induced pluripotent stem cell-derived neuronal cells, and we developed a transparent, single-chip device to fit seamlessly with a commercial headstage and amplifier. Using these optimized NEAs, we show that spontaneous, intracellular-like signals can be recorded from primary neurons, iPSC-derived neuronal networks, including CRISPR-engineered disease models of neuronal hyperexcitability, and iPSC-derived brain organoids during on-chip neuronal circuit formation. We also use pharmacological tools to confirm the validity of these recordings.

## Results

**Fig. 1:**
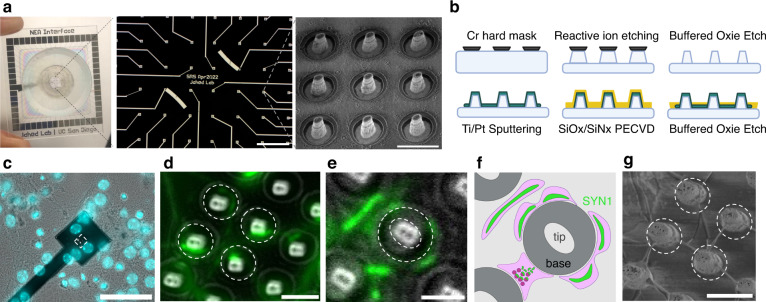
NEA fabrication and nanopillar interactions with neuron-like cells. **a** Schematic showing 4 cm × 4 cm NEA containing 60 total electrodes, each containing 9 nanopillars ~3 μm tall and 600 nm wide at the tip (scale bars: 500 μm, 5 μm). **b** Schematic summarizing NEA fabrication, consisting of two-step wet and dry etching and standard photolithography to pattern electrodes. **c** Representative fluorescence image with NucBlue staining of a single nanoelectrode showing sparse interactions between primary neuron somas and nanopillars (scale bar: 50 μm). **d** Merged fluorescence image, with the white dashed circles indicating 4 different nanopillars (scale bar: 3 μm), showing synapsin-1 punctae curving around separate nanopillars. **e** Another merged fluorescence image showing synapsin-1 punctae curving around a single nanopillar (scale bar: 2 μm). The white dashed circles indicate the tip and base of the nanopillar. This same pillar is depicted schematically in (**f**), demonstrating putative microscale curvature achieved by synapsin-1 distribution across the presynaptic region of the neurite. Created with BioRender.com. **g** Zoomed in SEM images of ROI shown in (**d**) with white dashed circles corresponding to the same four nanopillars (scale bars: 3 μm)

We used a lithography process with two steps of dry and wet etching to fabricate our NEAs (Fig. 1a, b). Our array included 60 electrodes (each electrode contains 9 nanopillars with an estimated conductive surface area of 56 μm^2^) organized into a square pattern. To test whether neuronal cell bodies or their neurites interact with these nanopillars, we plated primary hippocampal neurons from mice onto these electrode arrays and flat surfaces. We observed that neurites, along with cell bodies, interface intimately with the nanopillars (Fig. 1c, d). Also, we found that presynaptic protein, synapsin-1^29^, accumulates at high curvature regions of the pillars (Fig. 1d–f). Moreover, we show that the punctae density and circularity occupied by synapsin-1 punctae on these nanopillar electrodes are not significantly different when compared with those found on flat surfaces (Suppl. Figs. S1,3). While previous studies demonstrated that curvature affects the accumulation of synaptic proteins^30^, this was observed at the nano- rather than micro-scale, and thus outside of our pillar’s spatial regime. Nevertheless, further biological studies using 3D reconstructive imaging techniques should clarify how protein redistribution at the molecular level could scale to micron-sized structures^31,32^. In contrast, neuronal cell bodies tend to accumulate at the top of the pillars (Suppl. Fig. S2), rather than folding over them as previously reported. This might be due to differences in the diameter of the tip and smaller pitch we used compared to previous studies. Furthermore, we used scanning electron microscopy to confirm that several neurites can project towards single nanopillars (Fig. 1g). Collectively, these data indicate that nanopillar electrodes can interact with several primary neurites and occasionally with neuronal somas.

**Fig. 2:**
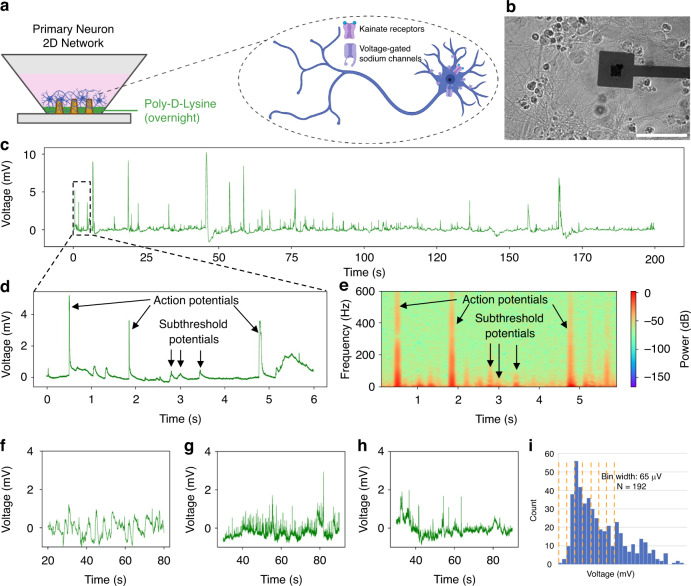
Intracellular-like supra- and sub-threshold recordings obtained from primary hippocampal neurons using NEAs. **a** Schematic showing coating protocol for seeding primary neurons on NEAs, as well as inset with schematic of targeted ion channels during pharmacological interrogation. Created in BioRender. Lab, B. (2023) B. **b** Brightfield image of neuronal network on top of NEA. **c** Representative 200s-long trace showing spontaneous intracellular-like signals from a single channel (*n* = 4 separate seeding experiments with 3–4 devices per seeding round). **d** Zoomed-in trace showing representative supra-threshold “action potentials” and sub-threshold potentials. **e** Frequency spectrogram used to validate intracellular-like signals and discern supra- (>100 Hz) and sub-threshold potentials (<100 Hz). Recording before (**f**), and after kainate addition (**g**), and after TTX addition (**h**) to the media (*n* = 2 separate pharmacological experiments, with 3 devices per experiment). **i** Histogram summarizing all collected sub-threshold spikes (*N* = 192), showing putative quantization of subthreshold potentials

Next, we tested whether these nanopillars can monitor changes in electrical activity, particularly after gaining access to the intracellular compartment^22,33^. We seeded primary hippocampal neurons on the NEA (Fig. 2a, b) and observed spontaneous intracellular-like events for several hundreds of seconds (Fig. 2c). Previously, using similar devices for cardiomyocytes, we observed intracellular action potential spikes only after active electroporation via a short electric pulse. However, for all neuronal cell types described in this paper we observed “spontaneous” intracellular-like signals without any direct stimulus. We believe that this smaller, “passive electroporation” may originate from static charge release upon circuit completion with the amplifier^34,35^, which is sufficient to temporarily permeabilize the neuronal (but not cardiomyocyte) plasma membrane. This effect might be related to the cell types’ different membrane capacitance properties, due to each of their specialized sizes and morphologies^36,37^. Next, we hypothesized that the observed intracellular-like signals might be either supra-threshold action potentials or subthreshold excitatory or inhibitory postsynaptic potentials. To test our hypothesis, we compared the frequency components of these events. We reasoned that there should be a correlation between the amplitude of the spikes and their classification, i.e. supra-threshold APs should have much larger amplitudes than subthreshold spikes. However, we expected that the amplitude of our recordings might be influenced by attenuation factors for each nanoelectrode, including the specific electronic properties, as well as the electrical coupling between the neuronal subcompartment during the recording session. We sought to use previously reported methods to distinguish supra- and subthreshold spikes instead. Previously action potentials have been shown to occur in the 300 Hz–5 kHz range^38^, while subthreshold postsynaptic potentials occur at lower frequencies (<10–200 Hz)^9,10,39^. To visualize these profiles, we used a frequency spectrogram and were able to identify action potentials and postsynaptic potentials (Fig. 2d, e, Suppl. Fig. 4, see methods). Notably, some of the spikes which exhibited high frequency AP-like components also showed distorted waveform shapes, particularly across time. While typically the action potential lasts 2–5 ms, previously recorded action potentials recorded using differently-sized nanopillars have exhibited signal broadening due to losses in the transfer function between the cell and the amplifier, such as shunting due to parasitic capacitance^10,40^. We believe that these broad spikes therefore represent a neuron or neuronal compartment that does not completely engulf the nanopillar during the recording session. Spikes that may be action potentials but are excessively broad typically do not pass our filter for detecting action potentials, and thus get discarded during postprocessing.

We further postulated that pharmacology could be used to validate that our putative subthreshold postsynaptic potentials are indeed neuronal in origin. We used kainate, a glutamate receptor agonist that increases neurotransmitter-mediated neuronal activity (i.e., should increase both APs and EPSPs)^41–43^ and tetrodotoxin (TTX), which blocks voltage-gated sodium channels and some kinds of glutamate receptors and attenuates some neuronal activity (i.e., should abolish APs but preserve some EPSPs)^44–46^. We observed that kainate increased the frequency of both supra- and subthreshold events, while tetrodotoxin reduced, but did not abolish, spiking, confirming that these events required neuronal activity (Fig. 2f–h). We also analyzed the patterns of these positively deflecting intracellular-like signals. Previous studies have shown that the subthreshold postsynaptic potentials are quantized based on the amount of neurotransmitter released from the presynaptic terminal. This has been demonstrated not only using the patch clamp technique, where each “quantum” is presumed to correspond to a single synaptic release event^47,48^, but also using NEAs, where each “quantum” is presumed to be some multiple of the discrete synaptic release due to the attenuation from recording with an imperfect seal^9,10^. Based on the biological amplitude of APs (100 mV), we computed our expected attenuation factor based on the typical amplitude of the high-frequency, intracellular APs (3 mV) we recorded from our devices (and found 33% attenuation (100%/3)). Since EPSPs typically exhibit amplitudes of around 10 mV, we reasoned that the majority of high-frequency spikes occurring below 0.3 mV should be subthreshold EPSPs. Sure enough, when we searched within this amplitude range for evidence of discrete spike amplitudes, we also observed clusters similar to previous groups (Suppl. Fig. 5). We observed events with a quantal size of 130μV, validating our classification method (Fig. 2i). Taken together, these data show that our NEA can acquire intracellular subthreshold events, which have properties like previously identified postsynaptic potentials.

**Fig. 3:**
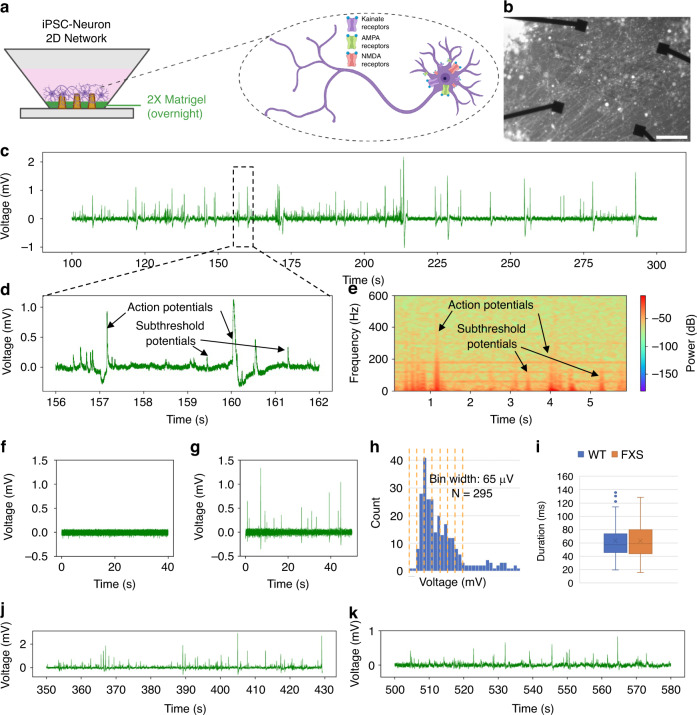
Intracellular-like supra- and sub-threshold recordings obtained from induced pluripotent stem cell-derived neuronal networks (iNeurons) using NEAs. **a** Schematic showing coating protocol for seeding iPSC neurons on NEAs, as well as inset with schematic of targeted ion channels during pharmacological interrogation. Created in BioRender. Lab, B. (2023) B. **b** Brightfield image of iPSC neuronal network on top of NEA. **c** Representative 200s-long trace showing spontaneous intracellular-like signals from a single channel (*n* = 4 separate seeding experiments with 3 devices per seeding round). **d** Zoomed-in trace showing representative supra-threshold “action potentials” and sub-threshold potentials. **e** Frequency spectrogram used to validate intracellular-like signals and discern supra- (>100 Hz) and sub-threshold potentials (<100 Hz). Recording before (**f**), and after (**g**) glutamate addition to the media (*n* = 2 devices). **h** Histogram summarizing all collected iPSC neuron spikes, showing putative quantization of subthreshold potentials (*N* = 295). **i** Box and whisker plots showing slight increase in duration of hyperexcitable iNeuron genotype when comparing (**j**) wildtype, (**k**) and Fragile X mutated iNeurons

We then tested whether our NEAs can also monitor activity changes in neurons derived from human induced pluripotent stem cells (iPSCs). Cellular overexpression of the pro-neural transcription factor Neurogenin-2 (NGN2) – to promote differentiation of iPSCs into excitatory neurons – is a popular and robust model to study neurological disease and development of neuronal networks^49–51^. Since these human iPSC-derived neurons have different properties compared to rodent primary hippocampal neurons, we modified our seeding procedures to obtain a similar dense network neurites (Fig. 3a, b). We observed that these neurons also exhibited APs and subthreshold events like primary neurons (Fig. 3c–e). Next, we added the excitatory neurotransmitter glutamate to transiently excite these NGN2-induced iNeurons^52^, and found that these neurons showed a transient increase in frequency of spikes consistent with previous results (Fig. 3f, g). Furthermore, we examined whether our NEA recordings can be used to distinguish between neurons derived from individuals with a disease diagnosis compared to controls. We plated wild-type, iPSC-derived neurons as well as CRISPR-engineered FMRP-deficient iPSC-derived neurons as a model for the Fragile-X syndrome (FXS) disease^53^. We observed an increase in spike durations in neurons derived from the later compared to the former (Fig. 3i–k). These data are consistent with previous studies which showed an increase in excitability of hippocampal neurons after silencing of the Fragile X Messenger Ribonucleoprotein 1 (FMR1)^54^. Collectively, these data suggest that NEAs can monitor neuronal activity from iPSC-derived neurons.

**Fig. 4:**
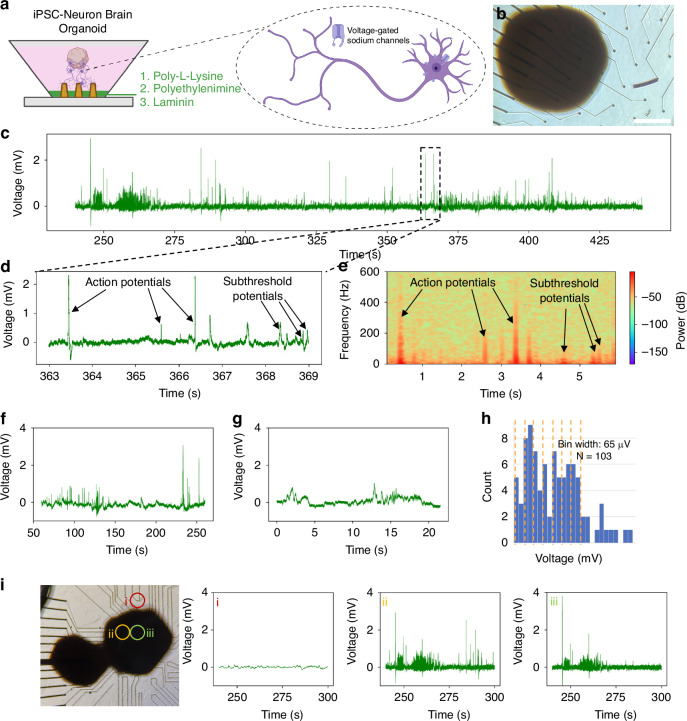
Supra- and sub-threshold intracellular-like recordings from outgrowth of induced pluripotent stem cell-derived brain organoids. **a** Schematic showing coating protocol for seeding iPSC-derived brain organoids on NEAs, as well as inset with schematic of targeted ion channels during pharmacological interrogation. Created in BioRender. Lab, B. (2023) B. **b** Brightfield image of brain organoids seeded on top of an NEA. **c** Representative 200 s-long trace showing spontaneous intracellular-like signals from a single channel (*n* = 2 separate seeding experiments with 3 devices per seeding round). **d** Zoomed-in trace showing representative supra-threshold “action potentials” and sub-threshold potentials. **e** Frequency spectrogram used to validate intracellular-like signals and discern supra- (> 100 Hz) and sub-threshold potentials (<100 Hz). Recording before (**f**), and after (**g**) TTX addition to the media (*n* = 2 devices). **h** Histogram summarizing all collected brain organoid spikes, showing putative quantization of subthreshold potentials (*N* = 103). (i) Bright field image showing organoids on an NEA, with intracellular recordings taken from overlying cells in (ii) and (iii) but no spiking from a bare electrode (**i**)

Finally, we used our NEAs to record neural activity from 3-dimensional iPSC-derived neuronal networks. Recent advances in organoid development have provided unparalleled access to 3-dimensional cultures of neuronal tissues, mimicking aspects of the brain and allowing for the discovery of many novel insights^55–57^. We found that our procedure for coating nanopillar electrodes needed to be changed to include a 24 h priming of the surface with poly-L-lysine and media before additional incubation with laminin and polyethylenimine (PEI) (Fig. 4a, b). We observed action potentials and subthreshold potentials in these 3-dimensional organoids (Fig. 4c–e). To confirm the biological origin of these events, we added tetrodotoxin and suppressed these responses (Fig. 4f–h). Moreover, we observed that our NEAs can be used to record intracellular events from overlying 3-dimensional cellular ensembles (Fig. 4i)

## Discussion

Our study shows that NEAs can be used to record electrical activity from young cultures of primary neurons, iPSC-derived neurons, and 3-dimensional organoids. Specifically, we observed action potentials and subthreshold events, which mimic postsynaptic potentials. We used frequency measurements and pharmacology to confirm that these events are distinct and are of biological origin. Qualitatively, we observe that the frequency profiles of supra- and subthreshold signals possess similar signatures across various neuronal cell types from different sources. We further observed that the “quantal” distribution of postsynaptic potentials can be discerned in NEA recordings from both 2D and 3D cytoarchitectures, which has not previously been shown. Furthermore, we demonstrate that our device and cell culture pipeline are compatible with both healthy and diseased cell lines. In the future, we hope to perform side-by-side biological studies to correlate electrophysiological differences to differences in gene expression and cellular organization directly on top of our devices.

Synaptic potentials are integrated by the neuron, and if a critical threshold is reached, the neuron fires an action potential. Intracellular recordings from neurons allow mapping of both synaptic potentials and action potential firing, allowing investigation into neural computation. The “Integrate-and Fire” model is widely used in computational neuroscience to model neural dynamics^58^ and is now >100 years old. High-throughput, simultaneous recordings of synaptic potentials and action potentials across networks of hundreds of interconnected neurons may provide novel insights into neural dynamics. Upon scaling the number of electrodes, this system could provide a platform for testing the effects of pharmacological manipulation or genetic mutation on neuronal function at a scale that is difficult to achieve with current approaches.

As we have shown in this study, neurites appear to preferentially interface with micro-pillars, allowing for direct intracellular recordings from the dendrites and axons of neurons. Dendrites are the location of most synaptic inputs on a neuron, so the ability to record from them directly, rather than the soma, is of great value. While most patch-clamp electrophysiology recordings target the soma, it is possible to reliably make recordings from dendrites as well, and there are various protocols available to this end^59,60^. However, this approach requires a highly skilled investigator and is most easily performed in neurons with large dendrites or proximal neurites.

The ability to repeatedly re-access the same cell for whole-cell recordings allows for within neuron controls, therefore increasing statistical power, while also allowing longitudinal recordings, on timescales relevant to key biological processes including synaptic plasticity and homeostatic mechanisms. While it is possible to perform multiple patch-clamp electrophysiology recordings from the same cell through the inclusion of fluorophores in the patch pipette^61,62^, these recordings were limited to ~1–2 neurons per brain slice. Two of the key limitations to this are the technical skills of the experimenter and their ability to patch sufficient neurons multiple times to allow for paired analysis.

The main limitations of the technology presented in this work include our lack of control over 1) the timing and duration of intracellular access, and 2) subcellular patterning of the neuronal network to control the spatiotemporal location of axons, dendrites, and somas with respect to the nanoelectrode during recording. Further studies involving longer recordings and longitudinal recordings over several days, while conducting membrane biophysical studies would clarify the exact mechanisms through which neuronal subcompartments are being “spontaneously” accessed. Additionally, micropatterning the NEA surface with extracellular matrix proteins would enable precise control over the distribution, guidance, and adhesion of neurons in certain patterns to serve as an additional validation step for supra- and subthreshold signals.

Future work to optimize our devices for use with brain slices would allow for recordings in intact neuronal networks for more translatable investigation. Furthermore, the data shown in this study includes a variety of different events, including intracellular and extracellular action potentials, and excitatory and inhibitory postsynaptic potentials. Future studies using targeted pharmacological intervention to selectively suppress certain events, such as blocking of synaptic transmission or action potential generation, while enhancing others would provide an opportunity to collect large amounts of data with which to generate algorithms for discrimination of different events to allow for more in-depth analysis.

## Methods

### Nanopillar chip and electrode array fabrication

Our fabrication process was adapted from previously described pipelines^22^. The nanopillar chips and the arrays were designed in Python using the phidl library. In the Nano3 Cleanroom (University of California, San Diego), 4-inch, fused silica wafers were cleaned in acetone and IPA with an N_2_ blow dry. KL 80/20 primer and AZ1512 positive photoresist were spun on according to the manufacturer’s recommendation. Circles were patterned using the MLA 150 maskless exposure tool and developed for 30 s using the AZ 400 K Developer. A Cr hard mask was deposited using the Temescal E-beam evaporation tool and excess Cr was lifted off with the RR41 remover. Reactive ion etching (Oxford Plasmalab 80) was used with Ar and CH_3_ gases to create 3 μm deep, tapered nanopillars with ~ 800 nm wide tips. The nanopillars were further etched isotropically with 20:1 buffered oxide etch (VWR, discontinued). At this point, wafers for nanopillar chips were diced and used for experiments. The following steps were performed on wafers to be used as NEAs. The same photolithography process as before was used to pattern the leads and electrode pads, although a longer development time (45 s) was used. 40 nm Ti and 100 nm Pt was sputtered onto the wafer using the Denton Discovery 635 tool, lifted off in RR41, and then passivated with alternating layers of SiO and SiN (Oxford Plasmalab PECVD). A stencil was used to prevent the passivation from covering the outer pads. Finally, the entire wafer was covered with photoresist and underwent an isotropic etch in 20:1 buffered oxide etch for 10 min to clear the passivation on top of the nanopillars, which etches first due to non-homogeneous photoresist thickness across the wafer. Finally, the photoresist was lifted off using RR41 remover and the wafers were diced.

### Synapsin-1 immunostaining and fluorescence imaging

Four nanopillar chips, each housed in a single 24-well plate containing primary hippocampal mouse neurons were fixed in 2.5% glutaraldehyde diluted in phosphate-buffered saline (PBS). After incubation in fixative solution for 30 min, a fresh solution was added, and the samples were kept overnight at 4 C. The next day, immunostaining was performed using an adapted protocol for the Synapsin-1 (D12G5) XP Rabbit mAb Alexa Fluor 488 Conjugate (Cat. no #13197, Cell Signaling Technology). Briefly, the samples were blocked for 1 h in blocking buffer containing 0.3% Triton-X 100 and 1% BSA diluted in 1x PBS. In the meantime, the synapsin-1 antibody conjugate was prepared at a dilution of 1:500 in a buffer containing 1% BSA, 0.3% Triton-X-100, and 1x PBS. After the blocking buffer incubation, the samples were washed two times in PBS, and incubated in the antibody solution for a minimum of 2 h at room temperature. The samples were then washed three times in PBS and incubated in a 1:200 dilution of Texas-red phalloidin and a 1:500 dilution of DAPI in PBS for an additional 30 min at room temperature. We then imaged the samples using a fluorescence microscope (Echo Revolution) with a 20x objective lens and a 60x water immersion lens.

### Synapse quantification using ImageJ

We loaded 60x maximum intensity projection z-stack images into ImageJ and performed the following process to threshold and count synaptic punctae. Images were converted to 8-bit greyscale, and the “Find Maxima” feature was used to threshold the images at 2600, and then the “Maxima within Tolerance”. Next, we used the “Set Measurements” feature to detect particles within a range of 0–0.5 um^2^ in size. We saved these measurements as.csv files and uploaded them to Python in Google Collaboratory for subsequent analysis across several images.

### Scanning electron microscopy

After fixation and immunostaining, samples were dehydrated using a serial dilution of ethanol, at 10, 30, 50, 70, 90, and 100% consecutively. Next, the samples were left to dry at room temperature overnight and imaged using the FEI Quanta SEM using 2 keV at high magnification. To perform SEM images of the same fields of view as fluorescence microscopy on the nanopillar chips, the nanopillars were counted starting from each corner of the nanopillar array.

### Electrochemical Impedance Spectroscopy (EIS)

We built a custom EIS setup in-house for NEA characterization. The device was connected to a potentiostat (PalmSens4) in the following configuration: The NEA device contained a well filled with phosphate-buffered saline (PBS). An aqueous Ag/AgCl electrode dipped into this bath served as the reference electrode, while at Pt/Ti also dipped into this bath served as the counter electrode. A titanium wire was taped to the outer pad of the nanoelectrode using electrical tape, which served as the working electrode. The following parameters were to conduct the measurement: an equilibration time of 3 s, fixed scan, an alternating current potential (E_ac_) of 0.25 V, a scan frequency with 52 frequencies ranging from 1 to 5000 Hz.

### Primary neuron culture

All animal procedures were performed in accordance with the Institutional Animal Care and Use Committee at the Salk Institute for Biological Studies. Briefly, pregnant C57BL/6 J mice were terminally anesthetized with carbon dioxide when embryos were E18. Hippocampal tissues were dissected in ice cold Hibernate E media containing 2% B-27 Supplement and 0.5 mM GlutaMAX. Hippocampi were incubated in TrypLE for 15 min at 37 °C. Excess TrypLE was discarded and titration media consisting of DMEM and 10% horse serum was added before triturating the cells with a pipette. Non-dissociated tissue was allowed to sink to the bottom before cells were seeded onto PDL coated NEAs at a density of 700 k cells per NEA or ~1000 cells/mm^2^. Cells were allowed to recover for 2 h in a 37 °C, 5% CO2 humidified incubator before fresh growth medium consisting of MACS Medium, 2% B-27 Supplement and 0.5 mM GlutaMAX was added. Half of the growth medium was changed every 3 days and on DIV3, 5uM Ara-C was added to induce cell death of non-neuronal cells. We performed primary neuron recordings with > 3 devices across 3 separate experiments on DIV5.

### iPSC-derived neuron seeding, differentiation, and culture

iPSC-neuron (iNeuron) recordings were performed with 2–3 devices each over five separate experiments. After several rounds of optimization, the coating which worked best for iPSC-derived neurons was a 2X dilution of Matrigel with overnight incubation at 37 °C. Two separate methods for plating iNeurons onto the devices were attempted. First, we attempted to differentiate iPSCs directly onto the NEAs. However, this resulted in a low density of differentiated iNeurons and a high number of undifferentiated iPSCs which diluted the recording measurements. The second method involved beginning the differentiation in well plates, and then replating onto the NEAs on DIV 8. Briefly, iPSCs were seeded onto 24-well plates. iPSCs were transduced with UNG lentiviral stock (Addgene plasmid #127288) and selected using a 500 ng/mL puromycin media change. The cells were expanded and reseeded into 6-well plates. On DIV 1, a half media change was performed using mTeSR Plus with 2 μg/mL doxycycline and 0.2X Rho-kinase inhibitor (RI), and on DIV2 without RI. On DIV3-7, another half media change was performed using Neurobasal-supplemented Neuronal Maintenance Medium (NMM) with 2 μg/mL doxycycline, 20 ng/mL BDNF, and 20 ng/mL GDNF. NEAs were sterilized and coated with 2x Matrigel for replating on DIV8, during which we used 2 mL Accutase for 1 well in a 6 well plate. Cells were centrifuged at 100 g and replated at 200,000 cells per NEA using plating media containing NMM, BDNF, GDNF, 2 μg/mLlaminin, 0.32 mg/mL papain, 0.05 mg/mL DNase, 5 mM MgCl_2_, and 1X RI. Half-media changes with NMM, BDNF, GDNF, and laminin were performed every 2–3 days until assay endpoint.

### iPSC-derived brain organoid seeding and culture

NEAs were cleaned with a fresh solution of 1% Terg-a-zyme for at least 2 h at room temperature. Next, the Terg-a-zyme was aspirated and NEAs were washed three times with autoclaved deionized water. The water was aspirated, and 70% ethanol added for 30 min inside the biosafety cabinet, followed by complete aspiration of the ethanol and three washes with autoclaved deionized water. The water was aspirated, and the device left to dry. After drying, 0.1% Poly-L-Lysine or media was added and incubated overnight either at 4 °C or inside the incubator, respectively. Next, the PLL/media was aspirated completely and 50 ml of PEI-filtered solution was added for 1 h inside the incubator. During incubations with small volumes of liquid, we also added a well filled with water next to the device to prevent evaporation of the solution. The solution was aspirated completely, washed three times with sterile, deionized water, and then the devices were left to dry at room temperature inside the biosafety cabinet. Thirty minutes prior to the secondary coating, an aliquot of laminin was thawed and diluted at a 1:25 ratio in medium. 50 ml of laminin was added to the center of the NEA and left for 1 h inside the incubator. Two to three organoids per NEA were seeded immediately after laminin incubation, and media was changed once a week before recording. We performed brain organoid recordings across three devices over two separate experiments. Recordings were performed 7 or 8 days after seeding, when neurite outgrowth from the organoids could be seen using a brightfield microscope with a 10x objective lens.

### Electrophysiology using MCS equipment

We performed multielectrode array recordings using the MultiChannel Systems (MCS) MEA2100 Mini System and MCS Experimenter Software. All recordings were conducted inside the incubator using the following procedure: The head stage, including the pins, a plastic cap (typically the lid of an empty pipette box), a sheet of heavy-duty aluminum foil, and the MCS pellet Ag/AgCl reference electrode were gently wiped down with 70% ethanol and placed inside of biosafety cabinet. Next, items were sterilized under UV light for between 10–30 min. The NEA was then removed from the incubator, manually loaded it onto the head stage, and the reference electrode added, inside the biosafety cabinet. The NEA was covered with the plastic cap, and the entire head stage was wrapped in Al foil, and then placed in the incubator. Typically, a separate incubator was set aside for devices which had already been recorded from, and for ongoing recordings. We then began data acquisition and recording ~5 min afterward. We used a sampling rate of 25 kHz, and the following hardware filters, which we adjusted using the MCS Config software: high pass cutoff at 0.1 Hz, and low pass cutoff between 3500 and 5000 Hz. If stimulation was used for electroporating specific electrodes, we used a biphasic square wave pulse of 3.5 V for 200 μs, followed by a second pulse of 1 V for 1000 ms. Files were exported to HDF5 format using the MCS Analyzer software, and then uploaded onto Google Drive for analysis.

### Pharmacology

Drug aliquots were prepared according to their solubility in either DMSO or water. Typical concentrations for each drug used were found in literature. Due to the time-sensitive nature of recording, the small volumes required for each device, and the lasting effect of the initial electroporation to the culture, drugs were thawed to room temperature and then 37 °C, added directly to the center of the culture by removing the desired volume inside the biosafety cabinet, opening the incubator door, quickly adding the droplet to 1 mL total volume of media, and then closing the door again. The table below summarizes the concentrations and volumes used for each drug described in this paper.

**Table:** 

Name of drug	Stock concentration	Volume added (μL)	Final concentration
Tetrodotoxin	500 μM	4	2 μM
Kainate	1 mM	2	2 μM
Glutamate	50 mM	50	2.5 mM

### Spike detection and plotting data

A bespoke spike detection algorithm was adapted from the MultiChannel Systems McsPyDataTools toolkit provided online. All recordings were uploaded to Google Drive, plotted, and analyzed using Python in Google Collaboratory. Specifically, the algorithm was modified to have a positive threshold for intracellular-like spikes instead of a negative one. Also, the data was filtered using an elliptic high pass filter with a cutoff of 70 Hz to get rid of low frequency noise and to stabilize the baseline for recording. Next, the positive threshold was set at five times the standard deviation of the high pass filtered signal itself, as previously reported in literature^9^. Spikes were detected as threshold crossings and converted into cutouts. From these cutouts, the spike amplitudes and durations were collected, and the data was converted to.csv files for subsequent analysis. Spikes were classified according to their frequency spectrogram and amplitude, where spikes having frequencies below 100 Hz and < 1 mV amplitude were considered to be postsynaptic potentials, while spikes having frequencies above 100 Hz and > 1 mV amplitude were considered to be action potentials. Postsynaptic potential histograms were created based on scatter plots comparing the maximum spike amplitude with other spike metrics such as total amplitude or duration. Based on these plots, the estimated quantal size of a single PSP was determined to be ~130 μV, and so the bin size was set to ~ 70 μV to create the histograms shown. The bin width and quantal size for the PSPs depends on the transfer function of the recording electrode, its amplifier, and the overlying neuron, as previously reported^9,10^.

## Supplementary information

Supplementary Information
