# Supplementary material for: Supra- and sub-threshold intracellular-like recording of 2D and 3D neuronal networks using nanopillar electrode arrays
Source: Microsyst Nanoeng. 2024 Dec 5;10:184. doi: 10.1038/s41378-024-00817-y (PMC11618331; doi:10.1038/s41378-024-00817-y)
Supplement: Supplementary file 1 — Supplementary Information [file 41378_2024_817_MOESM1_ESM.pdf]

Supra- and sub-threshold intracellular-like recording of 2D and 3D neuronal networks using nanopillar electrode arrays

Supplementary Information

Authors: Shivani Shukla[1,2], Joshua L. Schwartz[3,4], Callum Walsh[5], Wen Mai Wong[5], Vrund Patel[2], Yu-Peng Hsieh[1], Chichi Onwuasoanya[2], Shaoming Chen[2], Andreas Offenhäusser[6], Gert Cauwenberghs[1], Francesca Santoro[6,7], Alysson R. Muotri[3,7,8,9], Gene W. Yeo[3,4,10,11], Sreekanth H. Chalasani[5], Zeinab Jahed[1,2]*

[1]Shu Chien-Gene Lay Department of Bioengineering, University of California San Diego, La Jolla, California 92093, United States
[2]Aiiso Yufeng Li Family Department of Chemical and Nano Engineering, University of California San Diego, La Jolla, California 92093, United States
[3]Department of Cellular and Molecular Medicine, University of California San Diego, La Jolla, CA 92093, United States
[4]Institute for Genomic Medicine, University of California San Diego, La Jolla, CA 92093, United States
[5]Molecular Neurobiology Laboratory, The Salk Institute for Biological Studies, La Jolla, CA 92037, United States
[6]Institute of Biological Information Processing – Bioelectronics, IBI-3, Forschungszentrum Jülich, Jülich, 52428, Germany
[7]Neuroelectronic Interfaces, RWTH Aachen, Aachen, 52074, Germany
[7]Department of Pediatrics, University of California San Diego, San Diego, CA, United States
[8]Center for Academic Research and Training in Anthropogeny (CARTA) and Archealization (ArchC), University of California San Diego, La Jolla, CA, 92093, United States
[9]Sanford Stem Cell Education and Integrated Space Stem Cell Orbital Research (ISSCOR) Center University of California San Diego, La Jolla, CA, 92093, United States
[10]Sanford Stem Cell Institute Innovation Center, University of California San Diego, La Jolla, CA 92093, United States
[11]Center for RNA Technologies and Therapeutics, University of California San Diego, La Jolla, CA 92093, United States

*Address for correspondence: Z.J. (Zjahed@ucsd.edu)

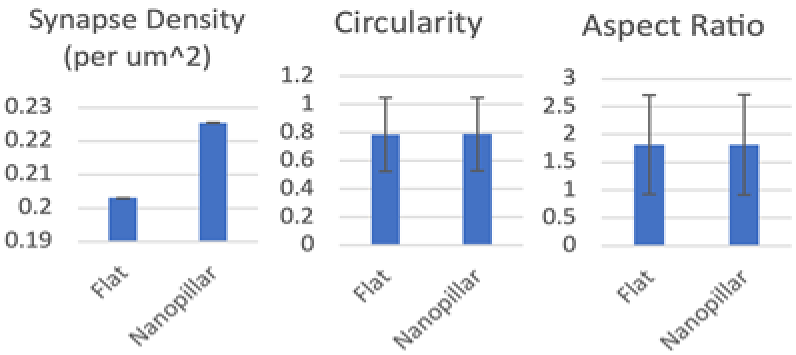

Figure S1. Summary of synapsin-1 metrics across flat and nanopillar surfaces.

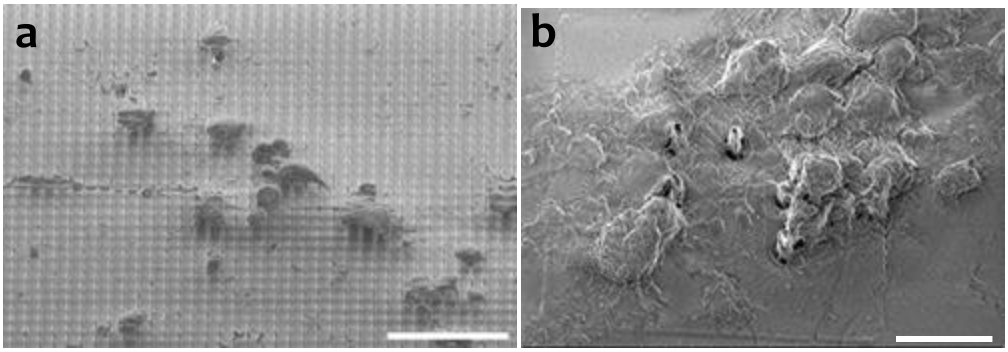

Figure S2. (a) Representative SEM image showing iPSC-derived neurons seeded on top of a “sea” of nanopillars, showing sparse interactions with iNeuron soma. Scale bar: 30 μm (b) Representative SEM image showing iNeurons interacting with a single nanoelectrode missing 3 pillars, showing sparse soma interactions. Scale bar: 10 μm.

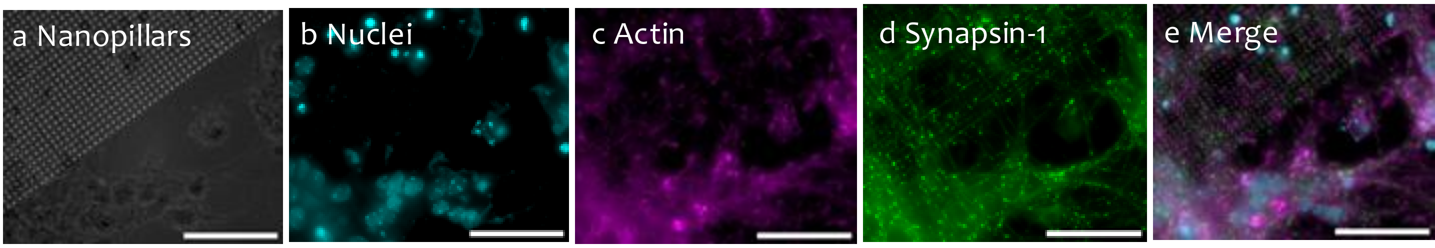

Figure S3. (a - e) Representative immunofluorescence images showing nanopillars, nuclei, actin, and synapsin-1 in separate channels (scale bars: 50 μm).

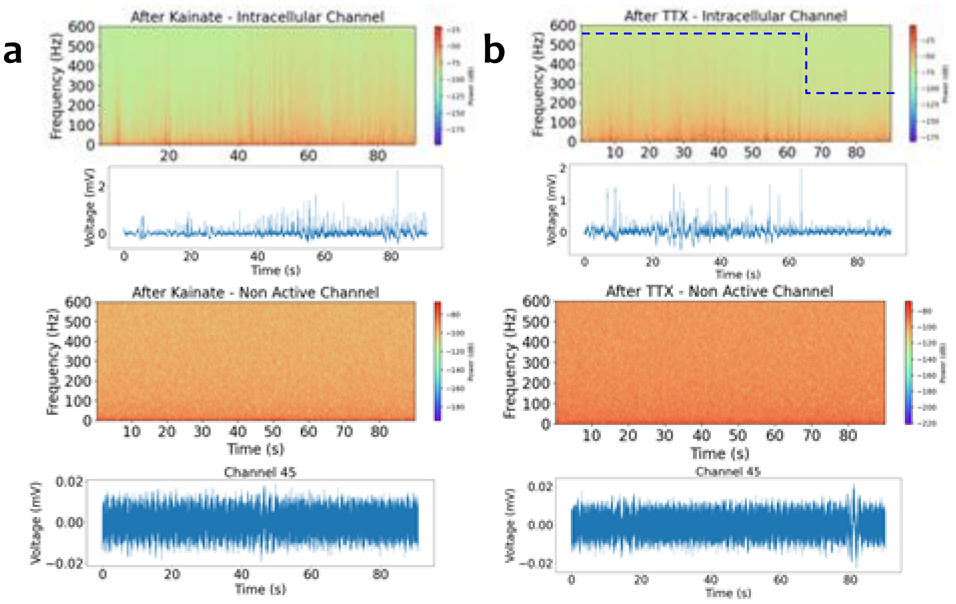

Figure S4. (a) Example intracellular and non-active (noisy) channels immediately after kainate addition, showing increased supra- (> 200 Hz) and sub- (<200 Hz) threshold activity only in the intracellular channel. Note that the downward spikes in the non-active channel represent 60 Hz electronic noise. (b) Example intracellular and non-active (noisy) channels immediately after tetrodotoxin addition, showing abolished supra- (> 200 Hz) and reduced sub- (<200 Hz) threshold activity only in the intracellular channel.

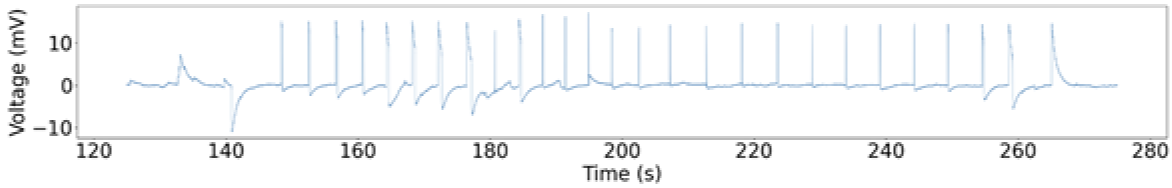

Figure S5. Recording trace showing “best” signals expected to have originated from a single neuron possessing tight coupling with a single nanoelectrode. The variations throughout the spike train suggest variable coupling over time, although the waveform shape, inter-spike interval, and frequency profiles suggest that these signals are biological in origin.

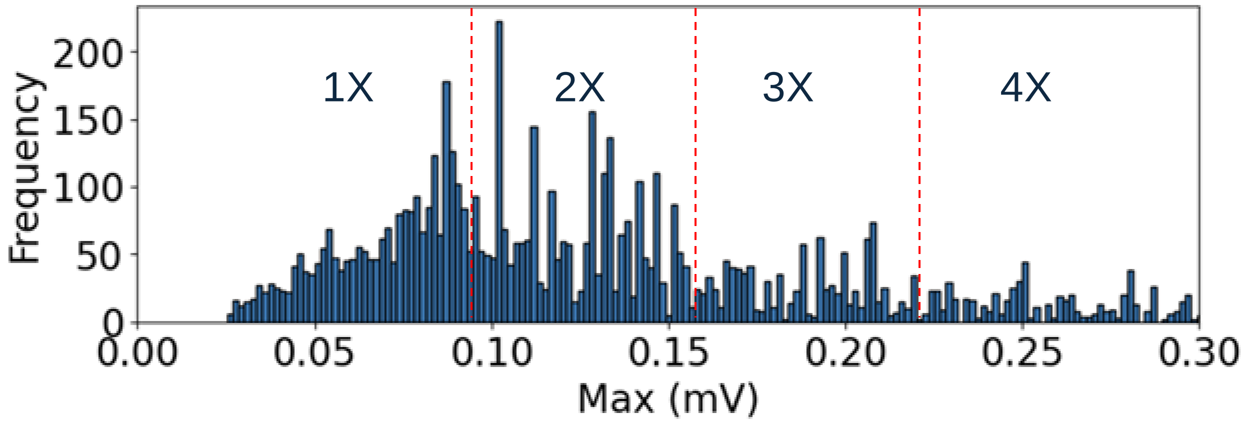

Figure S6. Histogram showing pooled data from all primary neuron spikes collected (N = 3270) across 4 different intracellular channels from one device during an entire recording session. This data was filtered above 70 Hz to eliminate any spikes from electronic noise, and spikes were detected using a threshold of 4x the standard deviation of the filtered signal. Metrics such as the maximum amplitude of the spike, the total amplitude, or even the minimum amplitude show similar "clustering" at rough multiples below 0.3 mV, the putative attenuated amplitude for subthreshold potentials.
